# 7-Dehydrocholesterol attenuates osteoarthritis by synergistically inhibiting oxidative stress, inflammation, and ferroptosis in macrophages

**DOI:** 10.3389/fphar.2026.1760112

**Published:** 2026-02-06

**Authors:** Wenchao Zhang, Mengru Hua, Xinyu Zhao, Guangxin Sun, Yuheng Pan, Le Miao, Xuzhuo Chen, Shanyong Zhang

**Affiliations:** 1 School of Stomatology, Shandong Second Medical University, Weifang, Shandong, China; 2 Graduate School, Dalian Medical University, Dalian, Liaoning, China; 3 Liaoning Provincial Key Laboratory of Oral Diseases, School and Hospital of Stomatology, China Medical University, Shenyang, Liaoning, China; 4 Shanghai Ninth People’s Hospital, College of Stomatology, Shanghai Jiao Tong University; National Clinical Research Center for Oral Diseases; Shanghai Key Laboratory of Stomatology; Shanghai Research Institute of Stomatology, Shanghai, China; 5 Department of Oral Surgery, Shanghai Ninth People’s Hospital, College of Stomatology, Shanghai Jiao Tong University, Shanghai, China

**Keywords:** 7-dehydrocholesterol, ferroptosis, inflammation, osteoarthritis, oxidative stress

## Abstract

**Objective:**

Osteoarthritis (OA) is a prevalent degenerative disorder affecting joints, characterized by progressive cartilage deterioration, inflammation of the synovium, and structural damage to subchondral bone. Inflammation mediated by synovial macrophages is a key driver of OA progression. Emerging evidence indicates that macrophage ferroptosis in inflamed synovium plays a pivotal role in disease advancement. 7-Dehydrocholesterol (7-DHC), an endogenous sterol with potent antioxidant properties due to its conjugated diene structure, effectively inhibits lipid peroxidation and ferroptosis. This study aimed to investigate whether 7-DHC delays OA progression by suppressing oxidative stress, inflammatory responses, and ferroptosis.

**Methods:**

To explore the mechanisms underlying inflammation *in vitro*, RAW 264.7 macrophages were stimulated using lipopolysaccharide (LPS). The effects of 7-DHC treatment were subsequently evaluated by measuring reactive oxygen species (ROS) production, levels of inflammatory cytokines, and expression of ferroptosis-related proteins including GPX4 and ACSL4. Reverse transcription quantitative polymerase chain reaction (RT-qPCR), immunofluorescence (IF), and Western blotting (WB) techniques were utilized to clarify the associated molecular pathways. Additionally, to verify the *in vivo* efficacy, an OA mouse model was established by administering complete Freund’s adjuvant (CFA) into the joint cavity, enabling assessment of inflammatory changes in synovial tissues and bone structural modifications following 7-DHC intervention.

**Results:**

The findings from RAW 264.7 macrophages stimulated with LPS indicated significant inhibition of ROS accumulation, downregulation of pro-inflammatory cytokines such as TNF-α and IL-1β, and normalization of ferroptosis-associated protein expression patterns after 7-DHC application. Additionally, 7-DHC markedly suppressed phosphorylation of MAPK/NF-κB pathway proteins while enhancing expression of Nrf2/HO-1 pathway proteins. *In vivo* experiments confirmed that 7-DHC significantly reduced inducible nitric oxide synthase (iNOS) expression in inflamed synovial tissue, promoted expression of GPX4, a key lipid peroxidation inhibitor, and improved the oxidative stress environment of synovial tissues. Consequently, knee joint inflammation and bone destruction were markedly alleviated in mice.

**Conclusion:**

7-DHC exerts anti-inflammatory and antioxidant effects by inhibiting the ROS/MAPK/NF-κB pathway and activating the Nrf2/HO-1 pathway. This reduces oxidative damage, inflammation, and ferroptosis in macrophages, thereby delaying OA progression. As a promising therapeutic strategy, 7-DHC may provide new research directions and clinical translational opportunities for OA treatment.

## 1 Introduction

OA is the most prevalent chronic degenerative joint disease worldwide. Its clinical manifestations mainly include joint pain, stiffness, and functional impairment, while pathological features encompass cartilage degeneration, synovial inflammation, and abnormal subchondral bone remodeling (Glyn-Jones et al., 2015; Musumeci et al., 2015). This condition predominantly affects individuals aged 60 years and older, with prevalence steadily rising due to global population aging (Felson and Hodgson, 2014; Hunter et al., 2014; Hunter and Bierma-Zeinstra, 2019). Current therapeutic strategies for OA primarily aim to alleviate symptoms and restore joint function. Typical pharmacological approaches include analgesics and nonsteroidal anti-inflammatory drugs (NSAIDs), complemented by non-pharmacological treatments such as ultrasound therapy, acupuncture, and thermal interventions. For patients with advanced OA, joint replacement surgery may be recommended (Buch, 2018). However, existing drug therapies cannot reverse disease progression and are often associated with gastrointestinal and cardiovascular adverse effects (Burmester and Pope, 2017; Fleischmann et al., 2017). The long-term efficacy of non-pharmacological treatments is also limited. Surgical interventions like total joint replacement are often not preferred by patients due to their invasive nature, uncertain long-term outcomes, and high economic costs (Glyn-Jones et al., 2015; Abramoff and Caldera, 2020). Therefore, developing novel treatment strategies that can slow OA progression with improved safety profiles represents an urgent research need.

Recent evidence suggests that OA is not simply a consequence of mechanical degeneration but represents a multifaceted immunometabolic condition characterized by the involvement of diverse cell populations and intricate molecular pathways (Abramoff and Caldera, 2020). Among these, synovial macrophages are key drivers of OA-associated inflammation. Once activated, these cells secrete substantial amounts of pro-inflammatory mediators, such as TNF-α, and iNOS, together with ROS. The resulting oxidative stress disrupts the joint microenvironment and precipitates chondrocyte apoptosis and degradation of the extracellular matrix (Chen et al., 2019; Deng et al., 2021). Ferroptosis not only contributes directly to cellular destruction but also intensifies inflammatory signaling, forming a self-reinforcing cycle of inflammation, oxidative stress, and ferroptosis that accelerates OA progression (Rochette et al., 2022; Al-Hetty et al., 2023).

At the mechanistic level, ferroptosis is modulated by several interconnected regulatory pathways. Glutathione Peroxidase 4 (GPX4) serves as a central antioxidant enzyme responsible for detoxifying lipid peroxides (Miao et al., 2022). Acyl-CoA synthetase long-chain family member 4 (ACSL4) facilitates the incorporation of polyunsaturated fatty acids into phospholipids, thereby enhancing cellular susceptibility to ferroptosis (Jiang et al., 2021). The transcription factor Nuclear Factor Erythroid 2-Related Factor 2 (Nrf2) preserves redox equilibrium by orchestrating the expression of antioxidant genes, glutathione (GSH) biosynthesis, and iron homeostasis (Ursini and Maiorino, 2020; Wang and He, 2022). Additional signaling cascades, including the mitogen-activated protein kinase (MAPK) and nuclear factor kappa-B (NF-κB) pathways, further contribute to the complex regulatory network governing ferroptosis (Liu-Bryan and Terkeltaub, 2015; Gaschler and Stockwell, 2017; El-Shitany and Eid, 2019). Consequently, therapeutic strategies aimed at modulating ferroptotic pathways have emerged as promising interventions for OA.

7-DHC, an endogenous intermediate in the cholesterol biosynthetic pathway, is abundant in human skin and serum (Göring, 2018). Owing to its distinctive conjugated diene structure, 7-DHC demonstrates strong phospholipid radical-scavenging activity within biological membranes and is considered a naturally occurring inhibitor of ferroptosis (Angeli et al., 2021; Li et al., 2024). Recent studies have shown that 7-DHC confers robust antioxidant and anti-ferroptotic effects in models of cancer (Lee et al., 2025), neurodegeneration (Tomita et al., 2022), and ischemia–reperfusion injury (Linkermann et al., 2014; Tonnus et al., 2021). Nevertheless, whether 7-DHC exerts protective effects in OA, characterized by chronic synovial inflammation and immune-metabolic dysregulation, remains unknown.

Therefore, we hypothesize that 7-DHC alleviates oxidative stress in macrophages and disrupts the vicious cycle of “inflammation-oxidative stress-ferroptosis” in OA pathology. It achieves this through synergistic regulation of the ROS/MAPK/NF-κB inflammatory axis and the Nrf2/HO-1 antioxidant axis. Specifically, 7-DHC dually regulates key ferroptosis proteins (e.g., ACSL4 and GPX4) at transcriptional and translational levels, providing multifaceted joint protection. To evaluate this hypothesis, we assessed the effects of 7-DHC on ROS production, inflammatory cytokine expression, and ferroptosis-associated markers using integrated *in vitro* and *in vivo* approaches to clarify underlying molecular mechanisms. Furthermore, we utilized a murine model of OA to investigate the protective actions of 7-DHC on joint tissues, aiming to provide novel mechanistic insights and assess its therapeutic potential in OA treatment.

## 2 Materials and methods

### 2.1 Reagents and antibodies

Materials and assay reagents utilized in the experiments encompassed lipopolysaccharide (LPS; extracted from *Escherichia coli*) procured from InvivoGen (San Diego, USA), α-MEM medium obtained from HyClone (Logan, USA), fetal bovine serum (FBS) supplied by Avantor (Ridley Park, USA), and penicillin–streptomycin acquired from Gibco (Gaithersburg, USA). Additionally, Beyotime Biotechnology (Shanghai, China) supplied the Cell Counting Kit-8 (CCK-8), fluorescent dyes including 4′,6-diamidino-2-phenylindole (DAPI), 2′,7′-dichlorodihydrofluorescein diacetate (DCFH-DA), MitoSOX™ Red, and Hoechst 33342 used for cellular assays. Dihydroethidium (DHE) was acquired from MedChemExpress (Shanghai, China). BODIPY™ 581/591 C11 was obtained from Invitrogen (Carlsbad, USA). The FerroOrange kit was purchased from Dojindo (Kumamoto, Japan). PrimeScript RT kit and SYBR® Premix Ex Taq™ II were acquired from Takara Bio (Otsu, Japan). The ferroptosis inducer RSL3 (HY-100218A) and inhibitor Ferrostatin-1 (HY-100579) were purchased from MedChemExpress (Monmouth Junction, USA).

Antibodies: Anti-HO-1 (AF5393) and NQO1 (DF6437) were purchased from Affinity Biosciences (Cincinnati, USA). Anti-phospho-p65 (Ser536, #3033), anti-p65 (#8242), anti-p38 (#8690), anti-phospho-p38 (Thr180/Tyr182, #4511), anti-ERK1/2 (#4695), anti-phospho-ERK1/2 (Thr202/Tyr204, #4370), anti-SAPK/JNK (#9252), anti-phospho-SAPK/JNK (Thr183/Tyr185, #4668), and anti-GAPDH (#5174) were obtained from Cell Signaling Technology (Danvers, USA). Anti-GPX4 (ab125066), anti-ACSL4 (ab155282), and anti-iNOS (ab283655) were acquired from Abcam (Cambridge, UK).

7-DHC was purchased from Sigma-Aldrich (#30800) and dissolved in dimethyl sulfoxide (DMSO) heated to 50 °C to prepare a 5 mg/mL stock solution, which was stored at −20 °C. Working concentrations were freshly prepared by diluting the stock solution with complete culture medium immediately before experiments. The final DMSO concentration in all 7-DHC working solutions did not exceed 0.1% (v/v). As DMSO at this concentration has been extensively shown to be non-toxic and non-interfering to cells, no dedicated solvent control group was included in this study (Sangweni et al., 2021). Untreated cells (Control group) served as the experimental baseline.

### 2.2 Cell sources and culture

The Cell Bank at the Chinese Academy of Sciences was the source for the murine macrophage cell line RAW 264.7. At 37 °C in a humidified environment with 5% CO_2_, cells were grown in α-MEM medium that also contained 10% FBS and 1% penicillin-streptomycin. The cells were passaged after they reached 80%–90% confluency. Pipetting or scraping cells gently harvested them to reduce cellular stress from enzymatic digestion.

### 2.3 Cell viability assay

In order to conduct viability tests, 96-well plates were seeded with 1 × 10^4^ cells per well using RAW 264.7 macrophages in logarithmic growth. After the cells had adhered, different amounts of 7-DHC (ranging from 0–400 μg/mL) were applied, and the cells were left to incubate at 37 °C for either 24 or 48 h. Following the incubation times, 10 µL of CCK-8 reagent was added to each well, and the cells were left to incubate for another 2 h in a dark environment. Following this, a microplate reader was used to record the absorbance at 450 nm. To ensure accuracy, blank wells devoid of cells were included in the baseline correction. As a percentage of the untreated control group, which was set at 100%, relative cell viability was computed.

### 2.4 Ferroptosis cell viability assay

To directly assess the inhibitory effect of 7-DHC on ferroptosis, RAW 264.7 cells were treated with RSL3 to induce ferroptotic cell death. Cells were seeded at 1 × 10^4^ cells/well in 96-well plates. After attachment, cells were randomly divided into six groups: Control, RSL3 model (1 μM), RSL3 + 7-DHC (20 μg/mL), RSL3 + 7-DHC (40 μg/mL), RSL3 + Ferrostatin-1 (1 μM, positive control), and 7-DHC-only (40 μg/mL). Cell viability was assessed 24 h post-treatment using the method described in 2.3 Section.

### 2.5 Detection of intracellular ROS and superoxide levels

RAW 264.7 cells were seeded at 5 × 10^4^ cells/well in confocal dishes. After attachment, cells were divided into three groups: Control, LPS (100 ng/mL), and LPS + 7-DHC (20 or 40 μg/mL). After 24 h, total ROS were detected using DCFH-DA, superoxide levels using DHE, and mitochondrial superoxide using MitoSOX Red. Probes were diluted 1:1,000 in serum-free medium and incubated for 20 min at 37 °C, protected from light. Cells were then washed twice with warm PBS. Staining with DAPI (1:1,000) for 5 min was done on the DCFH-DA group, whereas Hoechst 33342 (1:500) was used for 10 min on the MitoSOX group. Using ImageJ, we were able to quantify the fluorescence intensity of the images recorded by the confocal laser scanning microscope (CLSM, Leica TCS-SP5).

To evaluate the direct effect of 7-DHC on macrophage oxidative stress, an additional experiment was performed. Briefly, RAW 264.7 cells were cultured under identical conditions and divided into two groups: Control (normal medium) and 7-DHC-only (40 μg/mL 7-DHC, without LPS). After 24 h, total ROS and superoxide anion levels were measured using DCFH-DA and DHE probes, respectively, following the previously described method.

### 2.6 Detection of intracellular Fe^2+^ levels

The identical treatment conditions described earlier were applied to RAW 264.7 cells, which were seeded at 5 × 10^4^ cells per well, in order to measure intracellular Fe^2+^ concentrations. Next, the cells were exposed to a 1:1,000 dilution of FerroOrange and left to incubate at 37 °C for 20 min without light after 24 h. The cells were washed with warm PBS after being treated with Hoechst 33342 (1:500) for 10 min after FerroOrange staining. The imaging was done using CLSM, and the fluorescence intensity was measured using ImageJ software.

### 2.7 Lipid peroxidation assay

The treatments indicated before were applied to RAW 264.7 macrophages that were grown at a density of 5 × 10^4^ cells per well. Following a 24-h incubation period, the level of lipid peroxidation was measured using BODIPY™ 581/591 C11 (1:1,000). Following two washes with PBS, cells were imaged fluorescence using CLSM. We used ImageJ software to quantify the fluorescence intensity.

A commercial MDA assay kit from Nanjing Jiancheng (China) was used to quantify MDA, a crucial biomarker of lipid peroxidation. RAW 264.7 Cells in 6-well plates were grown for 24 h with or without different amounts of 7-DHC (0–40 μg/mL) and subjected to 100 ng/mL of LPS. Cells were sonicated in accordance with the manufacturer’s instructions after collection by scraping. Cell lysates were mixed with test reagents, then subjected to a 40-min heating cycle at 95 °C. After cooling, they were centrifuged at 10,000 × *g* for 10 min at 4 °C. Then, to measure the concentration of MDA, the supernatants were collected and their absorbance at 532 nm was measured.

### 2.8 Immunofluorescence staining

The RAW 264.7 macrophages were placed in confocal culture dishes at a density of 5 × 10^4^ cells/well. Cells were divided into three groups after attachment: Control, LPS (100 ng/mL), and LPS + 7-DHC (20 or 40 μg/mL). Cells were left to incubate at 37 °C for a full day. After 30 min in 4% paraformaldehyde, they were rinsed with PBS. The permeabilization process was initiated with 15 min of 0.5% Triton X-100 and continued with 1 h of blocking with 3% BSA. Overnight at 4 °C, the primary antibodies GPX4, iNOS, ACSL4, and phospho-p65 (all at a concentration of 1:200) were incubated. After adding the secondary antibodies, they were left to incubate in the dark for 1 h. We used CLSM to capture images of the cells, then ImageJ to measure the intensity of the fluorescence.

### 2.9 Quantitative real-time PCR

In 6-well plates, 5 × 10^5^ cells per well were used to seed RAW 264.7 macrophages in order to assess mRNA expression. After connecting, cells were exposed to either a control medium, LPS alone, or LPS plus 7-DHC for 24 h. An RNA isolation kit (Axygen, USA) was used for total RNA extraction, and the resulting RNA was reverse-transcribed into cDNA. Using SYBR® Premix Ex Taq™ II, real-time quantitative PCR was carried out on an ABI 7500 instrument. Every 10 μL reaction contained the following components: 5 μL SYBR Green reagent, 3 μL nuclease-free water, 1 μL cDNA template, 0.4 μL forward and reverse primers, and 0.2 μL ROX Reference Dye II. The PCR cycler was set up with an initial denaturation phase at 95 °C for 30 s, followed by 40 cycles of 95 °C for 5 s and 60 °C for 30 s. The relative gene expression was determined using the 2^−ΔΔCt^ method, with GAPDH serving as an internal reference. Table 1 contains the primer sequences that were utilized in these research.

**TABLE 1:** Primer sequences for qPCR.

Gene	Forward primer (5′→3′)	Reverse primer (5′→3′)
*Gapdh*	AGG​TCG​GTG​TGA​ACG​GAT​TTG	TGT​AGA​CCA​TGT​AGT​TGA​GGT​CA
*Acsl4*	TTG​TGG​CGA​ACT​TCT​TCA​CG	TTG​CCG​AAG​AGC​ATT​GAC​AC
*Gpx4*	GCA​GGC​AGG​GAA​GAC​AAT​C	CAG​GCA​GCT​CGT​TAT​TCA​GG
*Fth1*	CAG​GAT​GGC​AAC​AAC​CGA​A	TGG​CTA​AAG​GTG​AAG​GCT​CA
*Il1b*	TCG​CAG​CAG​CAC​ATC​AAC​AAG​AG	AGG​TCC​ACG​GGA​AAG​ACA​CAG​G
*Mmp9*	CTG​GAC​AGC​CAG​ACA​CTA​AAG	CTC​GCG​GCA​AGT​CTT​CAG​AG
*Nos2*	CGT​TCC​TGG​AGG​TGC​TTG​A	TCTCGGGTGCGGTAGGTG
*Ptgs2*	TTG​AGT​GGG​AAG​AAC​TGG​C	GGT​TGA​GTT​CAT​CAG​TCT​AC
*Sat1*	GGA​GAA​GGC​TGA​GAA​GGA​CG	CCT​TGT​AGT​AGC​CGA​GGC​AG
*Tnf*	TTA​GAA​AGG​GGA​TTA​TGG​CTC​A	TTT​GCA​GAA​CTC​AGG​AAT​GGA​C

### 2.10 Molecular docking (MD)

The 3D molecular structure of 7-DHC was downloaded from PubChem and converted into PDB format via Open Babel software (v2.3.2). The crystal structures of selected target proteins (NF-κB p65, JNK, p38, ERK) were obtained from the RCSB Protein Data Bank. Ligand and receptor structures underwent preparation using AutoDockTools (v1.5.6), involving the addition of polar hydrogens, assignment of Kollman charges, calculation of Gasteiger charges, and determination of rotatable bonds for ligands. Structures were then saved in PDBQT format, and molecular docking was performed using AutoDock Vina software (v1.1.2). Docking grids were centered on the receptor active pockets and sized to include the entire binding site. Binding modes were analyzed using PLIP to identify key intermolecular interactions. Bound conformations were visualized with PyMOL (v2.5.0).

### 2.11 Western blot

After being seeded at 5 × 10^5^ cells/well in 6-well plates, RAW 264.7 macrophages were exposed to Control, LPS, or LPS + 7-DHC (20 or 40 μg/mL) for a duration of 24 h. Using protease inhibitor-containing RIPA buffer, cells were lysed on ice. The total protein was extracted from the supernatants after centrifuging the lysates at 12,000 × g for 15 min at 4 °C. In order to find the protein concentration, the BCA test was used. Proteins of the same amount were separated using 10% SDS-PAGE and then transferred to PVDF membranes with a pore size of 0.22 μm. The following antibodies were used: GAPDH, p65, p-p65, iNOS, HO-1, NQO1, JNK, p-JNK, ERK, p-ERK, p38, and p-p38 (1:1,000 each). The membranes were then blocked with 5% BSA for 1 h and incubated overnight at 4 °C. After the membranes were washed with TBST, they were incubated with the secondary antibodies for 1 h. Using GAPDH as the internal loading reference, protein bands were seen using the Odyssey V3.0 imaging system and their intensities were measured using ImageJ software.

### 2.12 Mouse knee arthritis model

Following rigorous national ethical norms, all protocols involving animal testing were authorized by the Animal Ethics Committee of Shanghai Ninth People’s Hospital, which is connected with Shanghai Jiao Tong University School of Medicine (Approval No.: SH9H-2024-A11-1). To create an arthritic model in mice, researchers used a concentration of 5 mg/mL of complete Freund’s adjuvant (CFA). Each of the four experimental groups, consisting of sixteen male C57BL/6 mice that were 8 weeks old, received a random assignment: Control, CFA alone, low-dose 7-DHC (125 μg/mL), and high-dose 7-DHC (250 μg/mL). All surgical and injection procedures were carried out under anesthesia to ensure animal welfare. Mice were anesthetized initially by inhaling 3% isoflurane mixed with oxygen, then maintained at 1.5%–2% isoflurane using a nasal cone. Following anesthesia, each mouse received an intra-articular injection of 20 μL saline (Control) or CFA into the right knee joint. After 3 days, mice in Control and CFA groups received saline injections, whereas mice in the two 7-DHC treatment groups were administered 20 μL 7-DHC every other day for a total of seven injections. At the conclusion of the study, mice were rapidly anesthetized using inhalation of 3%–4% isoflurane. After confirming unconsciousness, euthanasia was immediately performed via intraperitoneal injection of an overdose of sodium pentobarbital (150 mg/kg). Complete cessation of respiration and heartbeat confirmed successful euthanasia. Knee joints were then harvested, fixed with 4% paraformaldehyde, washed thoroughly, and preserved in 75% ethanol.

### 2.13 Micro-CT scanning

The samples of fixed knee joints were scanned using a micro-computed tomography machine (μCT-100, SCANCO Medical) with the following parameters: 70 kV voltage, 200 μA current, 300 ms exposure time, and 10 μm voxel resolution. There was an evaluation of quantitative markers such trabecular BV/TV, Tb.Th, Tb.Sp, and Tb.N after rebuilding.

### 2.14 Histological and immunohistochemical (IHC) analysis

Decalcification was carried out for 4 weeks in a 10% EDTA solution with a pH of 7.4, with solution replacement every 3 days. H&E and Safranin O-Fast Green stains were applied to prepared coronal slices (5 μm). Sections were blocked with serum, peroxidase, and antigen retrieval before being treated with GPX4 (1:500) or iNOS (1:500) overnight at 4 °C for IHC. Sections were washed, then treated with secondary antibodies, DAB stained, and hematoxylin counterstained. The slides underwent dehydration, cleaning, and mounting. We used a Leica DM4000B microscope to take these pictures. ImageJ was used to analyze the percentage of positively stained cells. OARSI and synovitis scores were calculated as previously described (Krenn et al., 2006; Pritzker et al., 2006).

### 2.15 Statistical analysis

All experimental steps were independently conducted at a minimum of three repetitions, with data shown as mean ± SD. GraphPad Prism (version 9.0) was utilized for statistical evaluation and preparation of graphs. Prior to statistical testing, data were verified for variance homogeneity and normal distribution. An unpaired two-tailed Student’s t-test was applied to compare two independent groups, whereas multiple-group comparisons employed one-way ANOVA followed by Tukey’s *post hoc* test, provided variance homogeneity criteria were satisfied. A p-value lower than 0.05 was considered indicative of statistical significance.

## 3 Results

### 3.1 Effect of 7-DHC on RAW 264.7 cell viability

**FIGURE 1:**
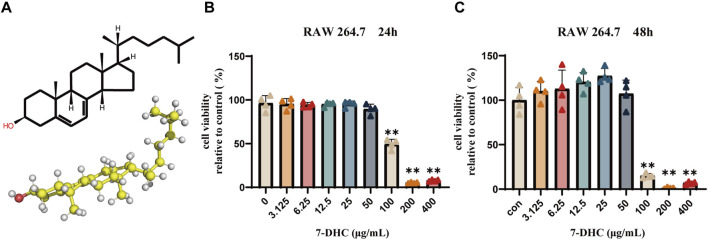
The chemical structure of 7-DHC and its effects on RAW 264.7 macrophages viability. **(A)** The chemical structure and ball-and-stick model. **(B)** Cell viability after 24 h of 7-DHC treatment (CCK-8 assay). **(C)** Cell viability after 48 h of 7-DHC treatment (CCK-8 assay). Data are mean ± SD (n = 3). *p < 0.05, **p < 0.01 vs. control, one-way ANOVA with Tukey’s *post hoc* test.

The molecular structure of 7-DHC is shown in Figure 1A. To evaluate potential cytotoxicity and determine a safe experimental concentration of 7-DHC, RAW 264.7 macrophages viability was measured by the CCK-8 assay after 24 and 48 h of treatment at various concentrations. At concentrations below 50 μg/mL, no significant cytotoxicity was observed, indicating good biocompatibility (Figures 1B,C). However, at 100 μg/mL, cell viability decreased significantly, especially after 48-h exposure. Therefore, to ensure normal physiological conditions, concentrations of 20 μg/mL and 40 μg/mL were selected for subsequent experiments.

### 3.2 7-DHC exhibits broad-spectrum ROS scavenging activity

**FIGURE 2:**
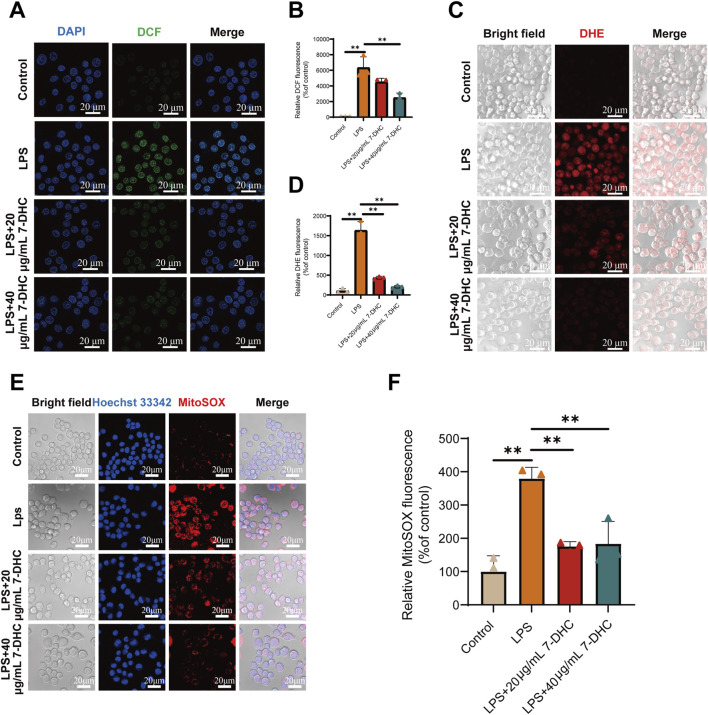
7-DHC attenuates LPS-induced intracellular ROS production in RAW 264.7 macrophages. **(A)** Representative fluorescence images of DCFH-DA staining. **(B)** Semi-quantitative analysis of DCF fluorescence intensity. **(C)** Representative fluorescence images of DHE staining. **(D)** Semi-quantitative analysis of DHE fluorescence intensity. **(E)** Representative fluorescence images of MitoSOX Red staining. **(F)** Semi-quantitative analysis of MitoSOX Red fluorescence intensity. Data are mean ± SD (n = 3). *p < 0.05, **p < 0.01 vs. control, one-way ANOVA with Tukey’s *post hoc* test.

To determine whether 7-DHC exerts antioxidant effects, intracellular ROS production was assessed in RAW 264.7 macrophages stimulated with LPS. Application of the fluorescent probe DCFH-DA indicated that LPS (100 ng/mL) treatment markedly enhanced intracellular ROS generation, as demonstrated by elevated fluorescence intensity. Treatment with 20 and 40 μg/mL 7-DHC significantly reduced fluorescence intensity in a dose-dependent manner (Figures 2A,B), demonstrating its effective scavenging of total ROS. Superoxide anion (O_2_^−^•) levels were detected using the DHE probe. LPS stimulation markedly increased intracellular O_2_^−^•, which was significantly reversed by 7-DHC treatment (Figures 2C,D).

Given mitochondria as a major ROS source, mitochondrial superoxide anion levels were measured using the MitoSOX Red probe. Results demonstrated that treatment with 7-DHC dose-dependently inhibited mitochondrial superoxide anion generation induced by LPS (Figures 2E,F). Collectively, these findings consistently indicate the potent antioxidant activity of 7-DHC against total ROS, superoxide anions, and mitochondrial superoxide, effectively alleviating oxidative stress in macrophages after inflammatory stimulation. Additionally, to further verify the intrinsic safety of 7-DHC, a monotherapy experiment was performed. As shown in Supplementary Figures S2C,D, compared to the control group, treatment with 7-DHC alone (40 μg/mL) did not provoke oxidative stress. Instead, it exhibited a trend toward further reducing baseline intracellular total ROS and superoxide anion levels. This indicates that 7-DHC may actively contribute to maintaining and optimizing the basal redox balance of macrophages, in addition to its potent ROS-scavenging capability under conditions of stress.

### 3.3 7-DHC exerts significant anti-inflammatory effects in macrophages

**FIGURE 3:**
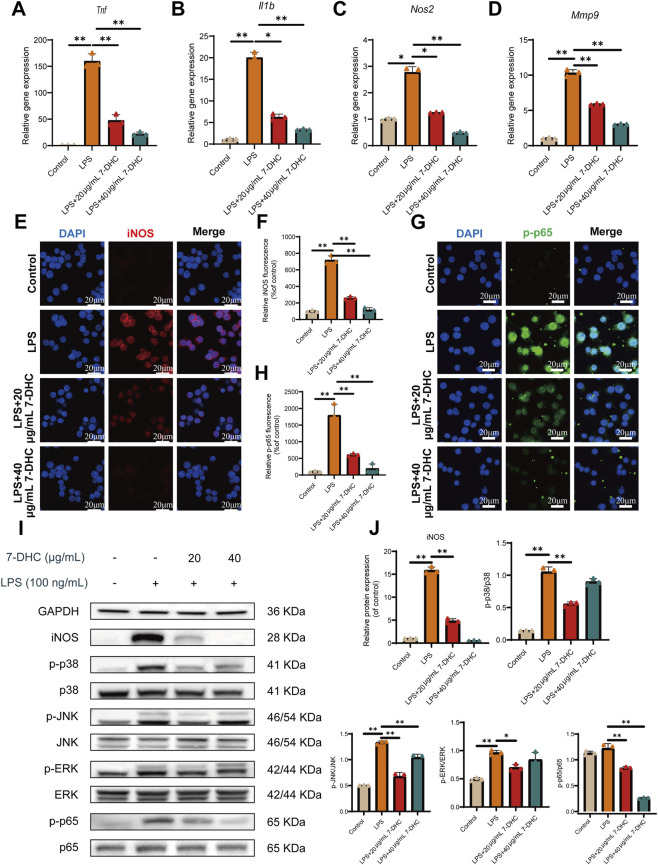
7-DHC Inhibits LPS-Induced Inflammatory Response in RAW 264.7 Macrophages. **(A–D)** RT-qPCR detection of mRNA expression levels for pro-inflammatory genes (*Tnf*, *Il1b*, *Nos2*, and *Mmp9*). **(E)** Representative image of iNOS immunofluorescence staining. **(F)** Semi-quantitative analysis of iNOS immunofluorescence intensity. **(G)** Representative image of p-p65 immunofluorescence staining. **(H)** Semi-quantitative analysis of p-p65 fluorescence intensity. **(I)** Western blot images showing protein expression of iNOS, p-p65, p38, p-p38, ERK, p-ERK, JNK, and p-JNK. **(J)** Quantitative analysis of Western blot results. Data are mean ± SD (n = 3). *p < 0.05, **p < 0.01 vs. control, one-way ANOVA with Tukey’s *post hoc* test.

Based on the established antioxidant properties, we further examined the regulatory effects of 7-DHC on macrophage inflammatory responses. Quantitative RT-PCR data indicated that LPS exposure significantly upregulated transcriptional expression of pro-inflammatory markers, including *Tnf, Il1b, Nos2,* and *Mmp9*, in RAW 264.7 cells (Figures 3A–D). Conversely, pretreatment with 7-DHC at concentrations of 20 and 40 μg/mL effectively suppressed these inflammatory genes in a dose-dependent manner, demonstrating substantial transcriptional inhibition.

To explore the structural basis of the anti-inflammatory effect, molecular docking simulations were conducted. Molecular docking, a computational technique for analyzing ligand–receptor interactions, typically indicates stronger affinity through more negative (lower) binding energies (Hsin et al., 2013). Docking results showed that 7-DHC binds specifically to active sites of key inflammatory signaling molecules, including NF-κB subunit p65 and MAPK pathway proteins (ERK, JNK, and p38), with binding energies all below −5 kcal/mol (ERK: −8.158 kcal/mol; JNK: −9.764 kcal/mol; p38: −9.131 kcal/mol; p65: −7.005 kcal/mol) (Supplementary Figure S1). Binding energies ≤ −7.0 kcal/mol indicate strong interactions (Pinzi and Rastelli, 2019). These findings suggest direct interactions of 7-DHC with these proteins, potentially interfering with their activation.

These computational results were further confirmed experimentally. IF staining and WB detected iNOS expression. LPS stimulation significantly increased iNOS fluorescence intensity and protein levels, which were dose-dependently reduced by 7-DHC (Figures 3E,F,I). NF-κB activation, characterized by p65 nuclear translocation, was analyzed by IF staining. LPS strongly promoted p-p65 nuclear accumulation, a process effectively inhibited by 7-DHC treatment (Figures 3G,H).

WB analysis (Figures 3I,J) further demonstrated increased phosphorylation levels of p65 (p-p65) after LPS stimulation, which were significantly suppressed by 7-DHC, consistent with the observed inhibition of p65 nuclear translocation. Similarly, LPS enhanced phosphorylation of MAPK pathway proteins (JNK, p38, ERK), while 7-DHC treatment reduced their phosphorylation to varying degrees.

Collectively, these data indicate that 7-DHC significantly suppresses the transcription of LPS-induced inflammatory mediators (TNF-α, IL-1β, MMP9) and downregulates iNOS expression at both gene and protein levels. Its anti-inflammatory mechanism likely involves direct inhibition of key signaling nodes within the NF-κB and MAPK pathways. These findings provide robust experimental support for the potential of 7-DHC as an anti-inflammatory agent.

### 3.4 7-DHC inhibits ferroptosis in inflammatory macrophages

**FIGURE 4:**
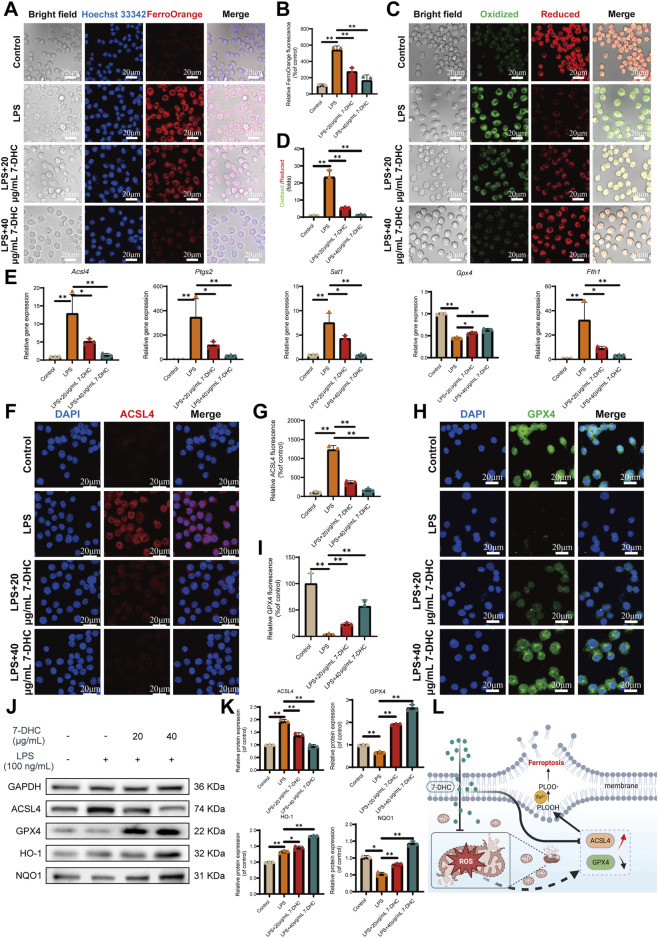
7-DHC inhibits LPS-induced ferroptosis in RAW 264.7 macrophages **(A)** Representative image of FerroOrange fluorescence staining. **(B)** Semi-quantitative analysis of FerroOrange fluorescence intensity. **(C)** Representative image of BODIPY C11 fluorescence staining. **(D)** Semi-quantitative analysis of BODIPY C11 fluorescence intensity. **(E)** mRNA expression analysis of ferroptosis-related genes (*Acsl4*, *Ptgs2*, *Sat1*, *Gpx4*, *Fth1*). **(F)** Representative image of ACSL4 immunofluorescence staining. **(G)** Semi-quantitative analysis of ACSL4 fluorescence intensity. **(H)** Representative image of GPX4 immunofluorescence staining. **(I)** Semi-quantitative analysis of GPX4 fluorescence intensity. **(J)** Western blot analysis of ACSL4, GPX4, HO-1, and NQO1 protein levels. **(K)** Semi-quantitative analysis of Western blot results. **(L)** Schematic diagram of the mechanism of 7-DHC-mediated inhibition of ferroptosis. Data are mean ± SD (n = 3). *p < 0.05, **p < 0.01 vs. control, one-way ANOVA with Tukey’s *post hoc* test.

Given the strong inhibitory effects of 7-DHC on oxidative stress, its role in LPS-induced ferroptosis in macrophages was further investigated. Initially, key biochemical markers associated with ferroptosis were measured. LPS treatment led to pronounced increases in intracellular MDA and Fe^2+^ accumulation compared with untreated controls (Figures 4A,B; Supplementary Figure S2). Pretreatment with various concentrations of 7-DHC substantially attenuated these elevations, indicating a dose-responsive inhibitory effect. These results suggest that 7-DHC may regulate iron metabolism by mitigating lipid peroxidation and iron accumulation triggered by LPS. Moreover, lipid peroxidation, a key characteristic of ferroptosis, was examined using the fluorescent probe BODIPY C11. LPS stimulation shifted fluorescence from red (reduced state) to green (oxidized state), signifying increased lipid peroxidation. Following 7-DHC treatment, green fluorescence decreased dose-dependently, while red fluorescence increased correspondingly (Figures 4C,D), confirming that 7-DHC significantly suppresses LPS-induced lipid peroxidation.

Next, core regulators of ferroptosis were examined at both gene and protein levels. RT-qPCR analysis revealed that LPS significantly elevated mRNA levels of ferroptosis-promoting genes (*Acsl4, Sat1, Ptgs2, Fth1*). In contrast, treatment with 7-DHC concentration-dependently reduced their abnormal expression. Meanwhile, GPX4, a critical negative regulator of ferroptosis, showed decreased expression under LPS stimulation, which was restored and enhanced by 7-DHC (Figure 4E). Corresponding changes at the protein level supported these findings. IF staining and WB collectively demonstrated (Figures 4F–K) that LPS increased ACSL4 protein expression while decreasing GPX4 protein levels, and these abnormal expression patterns were effectively reversed by 7-DHC. Additionally, WB analysis indicated that 7-DHC significantly elevated protein expression of HO-1 and NQO1, implying activation of the antioxidant Nrf2 signaling pathway (Figures 4J,K). Activation of Nrf2, together with restored expression of ferroptosis-regulating proteins (ACSL4 and GPX4), provides a comprehensive mechanistic basis explaining how 7-DHC alleviates oxidative stress, restores cellular redox equilibrium, and inhibits ferroptosis (Figure 4L).

### 3.5 7-DHC directly inhibits ferroptotic cell death induced by RSL3

To directly confirm the anti-ferroptotic activity of 7-DHC, RAW 264.7 macrophages were treated with RSL3, a selective GPX4 inhibitor, to induce ferroptosis. As illustrated in Supplementary Figure S2A, treatment with 1 μM RSL3 for 24 h markedly decreased cell viability compared with the control group, confirming successful ferroptosis induction. Co-treatment of cells with 7-DHC and RSL3 significantly reversed the reduction in viability in a dose-dependent manner. Notably, the protective effect of 40 μg/mL 7-DHC was comparable to that of the classical ferroptosis inhibitor, Ferrostatin-1 (Fer-1, 1 μM). Concurrently, 24-h of treatment with 40 μg/mL 7-DHC alone (“7-DHC only” group) resulted in no statistically significant differences in viability compared to untreated control cells. This finding indicates that 7-DHC itself exhibits no cytotoxicity at the effective dose tested. These data directly demonstrate that 7-DHC effectively inhibits ferroptotic cell death.

### 3.6 7-DHC improves knee OA in mice

**FIGURE 5:**
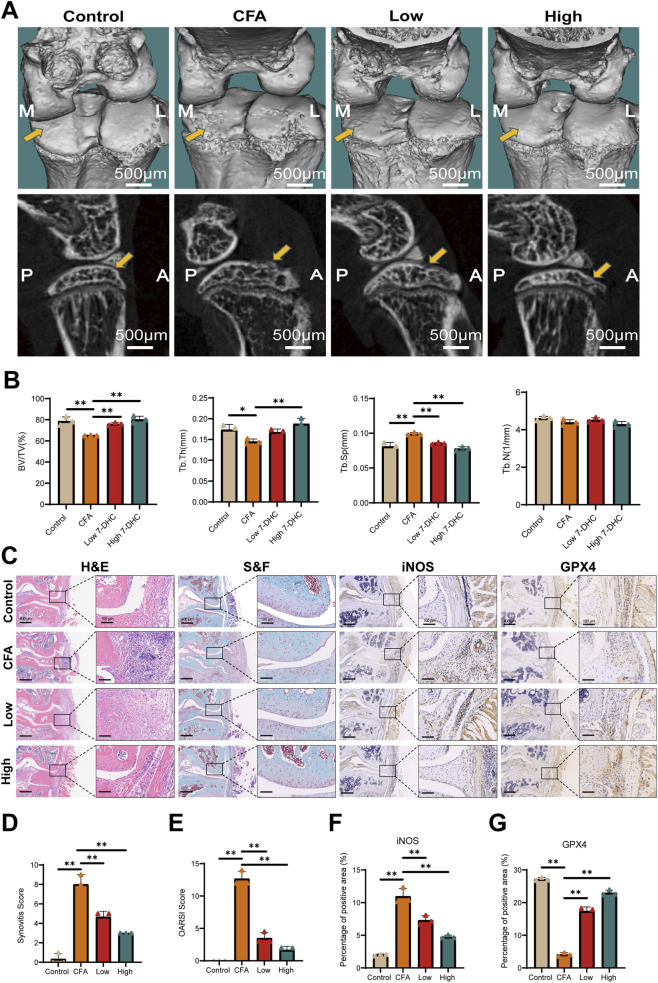
7-DHC Improves Knee Osteoarthritis in Mice. **(A)** Micro-CT scans and 3D reconstructed images of the knee joint. **(B)** Quantitative analysis of bone microarchitecture parameters: BV/TV, Tb.Th, Tb.Sp, Tb.N. **(C)** Representative images of H&E staining, S&F staining, and iNOS and GPX4 immunohistochemical staining in the knee joint. **(D)** Synovitis score. **(E)** OARSI score. **(F)** Semi-quantitative analysis of iNOS immunohistochemical intensity. **(G)** Semi-quantitative analysis of GPX4 immunohistochemical intensity. Data are mean ± SD (n = 3). *p < 0.05, **p < 0.01 vs. control, one-way ANOVA with Tukey’s *post hoc* test.

Following validation of the biosafety, antioxidant, anti-inflammatory, and anti-ferroptotic effects of 7-DHC *in vitro*, its therapeutic efficacy was further assessed using a mouse knee arthritis model induced by Complete Freund’s adjuvant (CFA). Micro-CT imaging and three-dimensional reconstruction revealed significant destruction of the articular surface, erosion of subchondral bone, and resorption pits in the CFA model group. Conversely, mice treated with 7-DHC, particularly at high doses, showed better preservation of joint structure and reduced bone erosion (Figure 5A). Quantitative analyses of bone microstructure parameters with CTan software (Figure 5B) demonstrated that 7-DHC significantly increased bone volume fraction (BV/TV) and trabecular thickness (Tb.Th), while reducing trabecular separation (Tb.Sp). These changes indicated improved trabecular density and preservation of bone mass. However, no significant differences in trabecular number (Tb.N) were detected among groups, suggesting that 7-DHC primarily enhances existing trabecular quality rather than quantity.

Histological examination provided further evidence supporting the protective effects of 7-DHC. Hematoxylin and eosin (H&E) and Safranin O–Fast Green (S&F) staining revealed that 7-DHC markedly decreased CFA-induced synovial inflammation and cartilage degradation. Additionally, IHC analyses showed that 7-DHC dose-dependently reduced synovial expression of the inflammatory mediator iNOS while increasing expression of the antioxidant enzyme GPX4 (Figures 5C,F,G). Consistent with histological findings, synovitis scores and OARSI scores were significantly lower in the 7-DHC treatment group (particularly at high doses) compared to the CFA model group, indicating effective alleviation of joint inflammation and delayed cartilage degeneration (Figures 5D,E). These findings suggest that 7-DHC synergistically regulates inflammation and ferroptosis in synovial tissues *in vivo*.

Overall, this study confirms that 7-DHC effectively alleviates synovial inflammation, inhibits cartilage matrix degradation, and improves bone microstructure damage in a CFA-induced knee OA model. These protective effects are closely related to its modulation of inflammatory responses and cellular redox balance within synovial tissue, highlighting the therapeutic potential of 7-DHC for OA treatment.

## 4 Discussion

OA is a chronic degenerative joint disease primarily characterized by cartilage degeneration, synovial inflammation, and subchondral bone remodeling (Berenbaum, 2013). Due to global population aging and rising obesity rates (Bliddal et al., 2014), OA prevalence continues to rise, representing a significant public health concern. The pathogenesis of OA is complex, involving various interconnected factors such as mechanical stress (Felson, 2013), and immune-inflammatory responses (Mobasheri et al., 2015; Orlowsky and Kraus, 2015; Mobasheri and Batt, 2016), ultimately causing joint structure damage and loss of function (Abramson et al., 2006). Despite advancements in understanding OA molecular mechanisms, targeted treatments effectively delaying disease progression remain limited.

Chronic inflammation in synovial tissues has been recognized as a central driver in OA progression (Roemer et al., 2011). Synovial macrophages, a key cell population in immune regulation, play an essential role in maintaining joint homeostasis and responding to local stimuli (Liu et al., 2024). Recently, oxidative stress was found to significantly accelerate OA progression (Rochette et al., 2022). Redox imbalance concurrently induces inflammatory responses and ferroptosis (Salzano et al., 2014; Galaris et al., 2019). Ferroptosis, an iron-dependent form of regulated cell death driven by excessive lipid peroxidation (Kagan et al., 2017), has been increasingly implicated in the pathological development of OA (Cui and Ye, 2024; Li et al., 2024). Consequently, mitigating oxidative stress within macrophages to suppress inflammation and ferroptotic injury has emerged as a promising therapeutic direction.

This study systematically examined the protective effects and underlying mechanisms of the endogenous sterol 7-DHC in OA using *in vitro* and *in vivo* experiments. As an endogenous intermediate of cholesterol biosynthesis, 7-DHC effectively captures free radicals through its conjugated diene structure, forming stable lipid hydroperoxides (LOOH). This interrupts lipid peroxidation chain reactions and inhibits ferroptosis (Li et al., 2024) (Figure 4L). Previous research has primarily explored the role of 7-DHC in cancer and ischemia-reperfusion injury. For example, inhibiting EBP (upstream enzyme) and blocking 7-DHC synthesis induces ferroptosis, suppressing tumor growth (Theodoropoulos et al., 2020). Conversely, inhibiting DHCR7 (downstream enzyme) elevates 7-DHC levels, promoting cancer metastasis but protecting against renal ischemia-reperfusion injury (Xiao et al., 2020; Mei et al., 2024; Li et al., 2025). However, the role of 7-DHC in OA, particularly its immunometabolic regulation in macrophages, has remained unexplored. Our study first demonstrates that 7-DHC alleviates OA progression via multiple mechanisms.

The findings demonstrated that macrophage ROS production induced by LPS could be effectively attenuated by 7-DHC, thereby alleviating intracellular oxidative stress and promoting stability within the cellular environment. RT-qPCR assays confirmed that 7-DHC markedly diminished the mRNA levels of key inflammatory markers, including *Tnf*, *Il1b*, and *Nos2*. Supporting these observations, WB and IF analyses showed pronounced reduction in iNOS protein levels. Given prior evidence indicating that ROS-triggered inflammation primarily activates MAPK (p38, ERK, JNK) and NF-κB (p65) signaling cascades (Berenbaum, 2004; El-Shitany and Eid, 2019; Liu et al., 2022), we next examined how these pathways were modulated by 7-DHC treatment. Western blot analysis revealed that 7-DHC significantly suppressed phosphorylation of key MAPK family proteins (ERK, JNK, p38) and NF-κB p65. Collectively, these findings highlight that the protective antioxidant and anti-inflammatory actions of 7-DHC are closely associated with its inhibitory regulation of ROS-mediated MAPK/NF-κB signaling. Additionally, 7-DHC treatment activated the Nrf2/HO-1 pathway, enhancing antioxidant enzyme expression. Notably, Nrf2, a central transcription factor for redox homeostasis, upregulates several enzymes involved in GSH synthesis and metabolism (Cuadrado et al., 2019). Since GSH is essential for GPX4 activity in scavenging lipid peroxides, activating Nrf2 maintains GPX4 function, thus protecting against ferroptosis. Moreover, Nrf2 activation partially inhibits NF-κB activity, possibly through ROS reduction or direct interaction with inflammatory pathways (Liu et al., 2018; Wang and He, 2022), resulting in a synergistic antioxidant and anti-inflammatory effect.

Iron overload is central to ferroptosis progression, ultimately leading to cell death via lipid peroxidation (Yan, 2024). This study evaluated ferroptosis by measuring MDA (a lipid peroxidation marker) and Fe^2+^ levels. 7-DHC significantly reversed the LPS-induced increase in these markers. Additionally, ACSL4 catalyzes the esterification of polyunsaturated fatty acids (PUFAs) into membrane phospholipids such as PE, a primary target of lipid peroxidation during ferroptosis (Ding et al., 2023). WB analysis confirmed that 7-DHC suppressed ACSL4 expression, reducing lipid peroxidation substrates. Concurrently, it increased the expression of GPX4, enhancing cellular resistance against lipid peroxidation. Thus, 7-DHC simultaneously modulates ACSL4 and GPX4 expression, directly validating its anti-ferroptotic mechanism. Importantly, direct functional assays further provide comprehensive evidence for the anti-ferroptosis activity of 7-DHC, spanning molecular mechanisms to cellular outcomes. Specifically, experiments demonstrated that 7-DHC effectively counteracts ferroptosis induced by RSL3, exhibiting efficacy comparable to Ferrostatin-1. This functional validation not only confirms 7-DHC as a potent ferroptosis inhibitor but also completes the evidence chain from molecular regulation (GPX4 upregulation) to improved cell survival. Additionally, at safe doses, 7-DHC actively reduces basal ROS levels, suggesting potential pre-regulatory effects on redox homeostasis.

**FIGURE 6:**
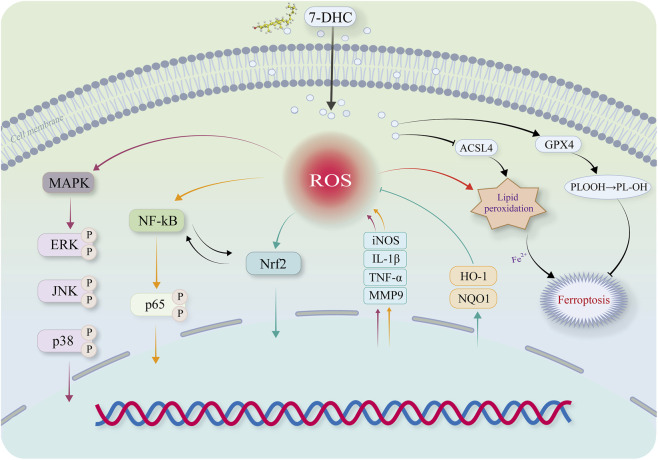
Schematic diagram of the mechanism by which 7-DHC inhibits macrophage inflammatory response and ferroptosis.

In summary, this study advances understanding of the protective role of 7-DHC in OA. It transitions 7-DHC from being merely a known ferroptosis inhibitor to a comprehensive protective agent capable of simultaneously modulating multiple macrophage-related pathways within the OA immunometabolic network. For the first time, this research systematically elucidates a triple synergistic mechanism of 7-DHC action in OA synovial macrophages: simultaneous suppression of the ROS/MAPK/NF-κB inflammatory axis, activation of the Nrf2 antioxidant pathway, and reshaping of ferroptosis regulatory networks involving ACSL4/GPX4 (Figure 6). This establishes a novel therapeutic paradigm targeting the complex pathological network of OA.

The efficacy of 7-DHC observed *in vivo* was validated in a CFA-induced mouse model of knee arthritis. Treatment significantly alleviated synovial inflammation and cartilage degeneration, as evidenced by lower synovitis and OARSI scores. Moreover, 7-DHC improved subchondral bone microarchitecture, reflected by increased BV/TV and Tb.Th, indicating effects beyond symptomatic relief toward true disease modification. Molecularly, 7-DHC reduced synovial iNOS expression and enhanced GPX4 levels, verifying its dual regulation of inflammation and ferroptosis *in vivo*. Although the acute CFA model differs from chronic human OA, it effectively mimics the core pathological mechanisms of the inflammation-oxidative stress-cell death axis. Thus, this study confirms the intervention capacity of 7-DHC in these fundamental OA drivers, highlighting its potential therapeutic value.

However, several limitations remain. First, the *in vivo* pharmacokinetic properties of 7-DHC are unclear. Further studies are necessary to evaluate its tissue distribution, metabolism, and long-term biosafety. Given the low bioavailability of 7-DHC, future investigations should explore novel delivery methods such as nanoparticles, transdermal patches, or targeted liposomes to enhance its joint-specific bioavailability and clinical applicability. Second, regarding mechanistic depth: Although strong evidence suggests macrophages as the primary targets, further confirmation through cell-specific knockout models is required. The precise interactions between pathways (e.g., Nrf2 and NF-κB) also warrant quantitative evaluation using specific agonists/inhibitors. Finally, validating long-term efficacy using chronic OA models (e.g., DMM) is essential for clinical translation.

In summary, this study demonstrates that 7-DHC corrects oxidative stress, ameliorates inflammatory responses, and inhibits ferroptosis in synovial macrophages by synergistically modulating the ROS/MAPK/NF-κB axis and activating the Nrf2/HO-1 pathway, ultimately delaying OA progression. The identified triple synergistic mechanism and disease-modifying properties position 7-DHC as an exceptionally promising candidate for OA therapy, offering substantial theoretical significance and translational potential.

## 5 Conclusion

In this study, we comprehensively evaluated the therapeutic actions and underlying mechanisms of 7-DHC in inflammation-related OA. Our findings demonstrate that 7-DHC attenuates oxidative injury, inflammatory responses, and ferroptosis in macrophages by inhibiting the ROS/MAPK/NF-κB signaling cascade while simultaneously activating the Nrf2/HO-1 antioxidant pathway, thereby highlighting its potential as a candidate intervention for OA management. In a CFA-induced mouse model, 7-DHC significantly improved joint inflammation, cartilage structure, and bone integrity. Collectively, these findings highlight 7-DHC as a promising therapeutic agent for OA, providing crucial experimental evidence and laying a foundation for further clinical research.

## Data Availability

The datasets presented in this study can be found in online repositories. The names of the repository/repositories and accession number(s) can be found in the article/Supplementary Material.
