# Supplementary material for: 7-Dehydrocholesterol attenuates osteoarthritis by synergistically inhibiting oxidative stress, inflammation, and ferroptosis in macrophages
Source: Front Pharmacol. 2026 Feb 6;17:1760112. doi: 10.3389/fphar.2026.1760112 (PMC12920527; doi:10.3389/fphar.2026.1760112)
Supplement: Supplementary file 1 [file DataSheet2.pdf]

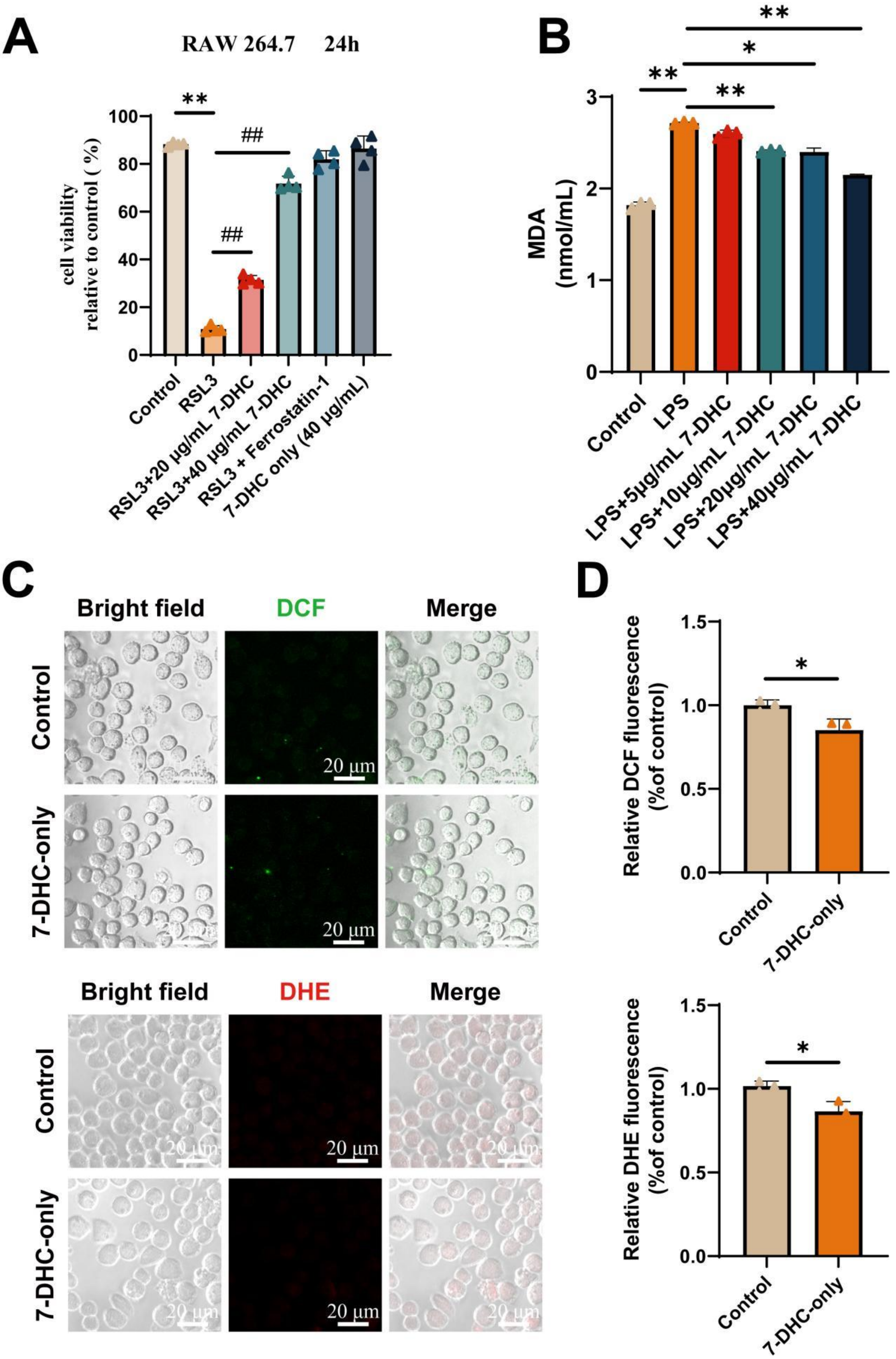
A
RAW 264.7 24h
cell viability relative to control (%)
0
20
40
60
80
100
**
##
##
Control
RSL3
RSL3+20 μg/mL 7-DHC
RSL3+40 μg/mL 7-DHC
RSL3 + Ferrostatin-1
7-DHC only (40 μg/mL)
B
MDA (nmol/mL)
0
1
2
3
**
*
**
**
Control
LPS
LPS+5μg/mL 7-DHC
LPS+10μg/mL 7-DHC
LPS+20μg/mL 7-DHC
LPS+40μg/mL 7-DHC
C
Bright field
DCF
Merge
Control
7-DHC-only
20 μm
Bright field
DHE
Merge
Control
7-DHC-only
20 μm
D
Relative DCF fluorescence (%of control)
0.0
0.5
1.0
1.5
*
Control
7-DHC-only
Relative DHE fluorescence (%of control)
0.0
0.5
1.0
1.5
*
Control
7-DHC-only

Fig. S2 7-DHC Directly Inhibits Ferroptosis and Improves Basal Redox State in Macrophages. (A) 7-DHC rescues RSL3-induced ferroptotic cell death. Cell viability was measured by CCK-8 assay after 24-hour treatment. (B) 7-DHC reduces MDA levels in LPS-stimulated macrophages. (C) Representative fluorescence images of DCFH-DA and DHE staining. (D) Semi-quantitative analysis of DCF and DHE fluorescence intensity. Data are mean ± SD (n=3). *$p < 0.05$, **$p < 0.01$ vs. Control group; #$p < 0.05$, ##$p < 0.01$ vs. RSL3 group in (A); one-way ANOVA with Tukey's post hoc test. *$p < 0.05$, **$p < 0.01$ vs. Control in (B) and (D); unpaired two-tailed Student's t test.
