# Supplementary figures and images for: 7-Dehydrocholesterol attenuates osteoarthritis by synergistically inhibiting oxidative stress, inflammation, and ferroptosis in macrophages
Source: Front Pharmacol. 2026 Feb 6;17:1760112. doi: 10.3389/fphar.2026.1760112 (PMC12920527; doi:10.3389/fphar.2026.1760112)

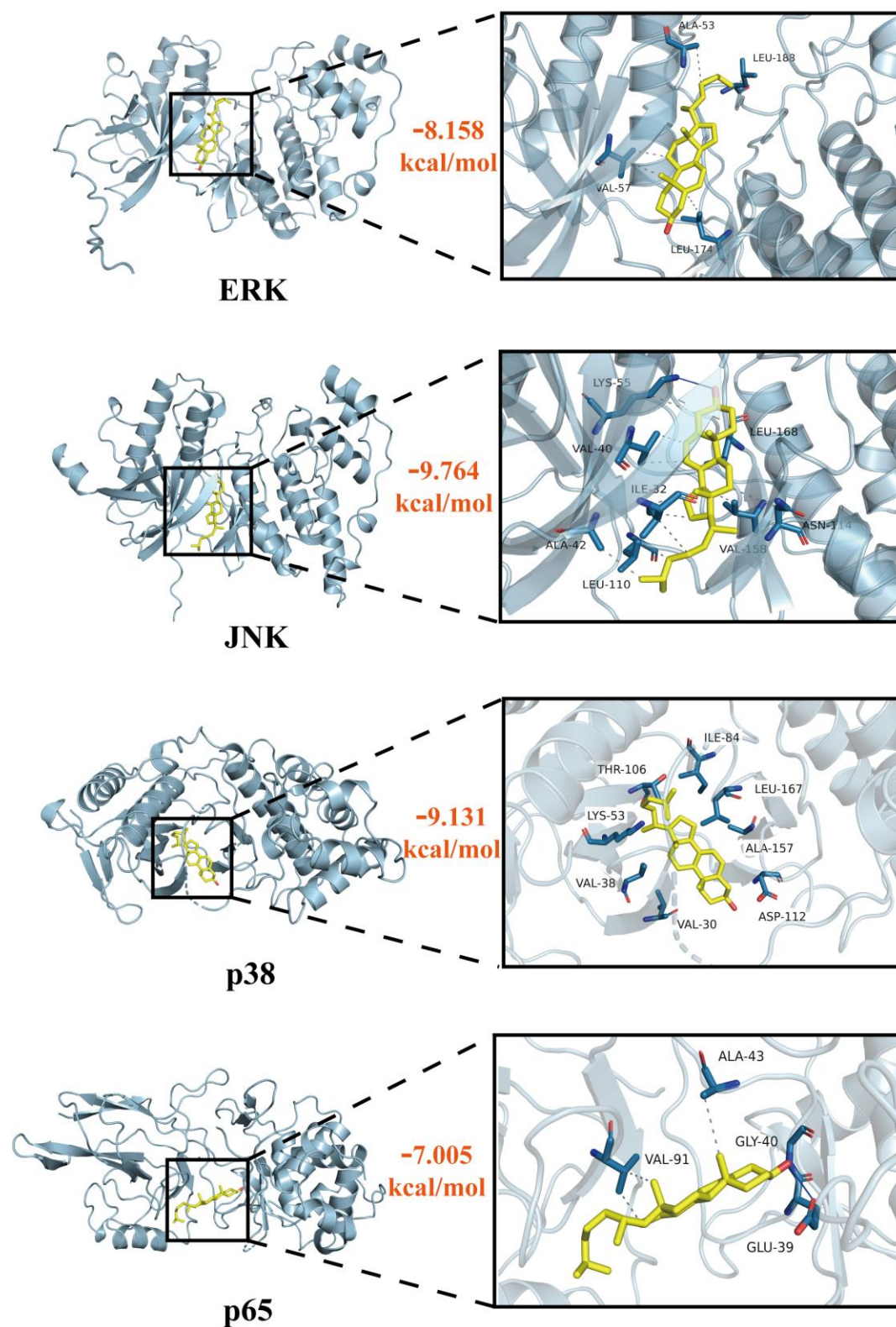

**Fig. S1** Molecular docking of 7-DHC with key signaling proteins(ERK, JNK, p38, p65).

Supplement: Supplementary file 2 [file DataSheet1.pdf]
